# Active management of the third stage of labor and associated factors among maternity care providers in public health facilities in Eastern Ethiopia: a multi-center study

**DOI:** 10.1186/s12884-023-06009-2

**Published:** 2023-09-30

**Authors:** Birhane Fissahaye, Merga Dheresa, Nega Assefa, Dejene Tesfaye, Addis Eyeberu, Bikila Balis, Adera Debella, Berhe Gebremichael, Tamirat Getachew

**Affiliations:** 1 School of Nursing and Midwifery, College of Health Science and Comprehensive Specialized Hospital, Aksum University, Aksum, Ethiopia; 2 School of Nursing and Midwifery, College of Health and Medical Sciences, Haramaya University, Harar, Ethiopia; 3 School of Public Health, College of Health and Medical Sciences, Haramaya University, Harar, Ethiopia

**Keywords:** Active management, Third stage labor, Maternity care provider, Eastern Ethiopia

## Abstract

**Introduction:**

The third stage of labor is the shortest, most critical, and hazardous stage as it is linked with postpartum hemorrhage, the leading cause of maternal mortality and morbidity. Postpartum hemorrhage can be prevented by 60% with active management of the third stage of labor (AMTSL). Few studies have been conducted in different parts of Ethiopia showing rates of AMTSL ranging from 16.7% to 43.3%. Limited information, however, exists about its practice in our study area. Thus, we aimed to assess the practice of AMTSL and associated factors among maternity care providers in public health facilities in eastern Ethiopia.

**Methods:**

An institution-based cross-sectional study design was used among 270 maternity care providers in public health facilities in eastern Ethiopia. They were recruited using cluster sampling techniques in their health facilities from July 15—October 30/2021. Pretested self-administered questionnaires and an observational checklist were used to collect data. Descriptive, binary, and multivariable logistic regression analyses were performed. Adjusted odds ratios with 95% confidence intervals were used for statistically significant associations.

**Results:**

Good practice of AMTSL occurred in 40.3% (95% CI: 34.5%—46.1%) of births. Being trained (aOR 3.02; 95% CI 1.60–5.70); presence of birth assistance (aOR 2.9; 95% CI 1.42–6.04); having the highest educational level (aOR 4.21; 95% CI 1.08–16.40); and having good knowledge (aOR 3.00; 95% CI 1.45–6.20) were factors statistically associated with maternity care providers’ good practice of AMTSL.

**Conclusion:**

Active management of the third stage of labor was practiced with low rates in the study area. Therefore, we suggest that the stakeholders could enhance the presence of birth assistance during all births and provide education to attain higher educational levels and continuously update the maternity care providers’ level of knowledge through comprehensive and on-the-job training to increase the good practice of the third stage of labor.

## Introduction

The third stage of labor (TSL) is the shortest, most critical, and hazardous stage of childbirth as it is linked with postpartum hemorrhage (PPH) [1–3]. Active management of the third stage of labor (AMTSL) is a package of interventions that entail measures aimed at facilitating the birth of the placenta by boosting uterine contractions and thereby preventing primary PPH [4, 5]. Administration of uterotonic drugs within 1 min after the birth of the newborn, controlled cord traction (CCT), to deliver the placenta and subsequent uterine massage every 15 min for the first two hours are the usual components of AMTSL [6, 7].

Administration of uterotonic drugs is the first component for the prevention of PPH and reduces the chance of PPH by 40% [8, 9]. The second component is applying CCT by a skilled birth attendant which is the first intervention for the management of retained placenta [6]. Compared to expectant care, CCT reduced PPH by nearly half [10]. Finally, in all laboring mothers immediate and subsequent massage of the uterine fundus after delivery of placenta and membranes every 15 min for the first two hours or until the uterus stays contracted well [11]. Massage is supposed to prevent bleeding by stimulating uterine contractions, potentially by activation of local prostaglandin production [12]. Most countries have poor rates of immediate fundal massage and follow-up palpation after placenta delivery, implying a lack of women's surveillance during the hours when the majority of maternal deaths occur [13].

PPH is the leading cause of maternal mortality and morbidity associated with uterine atony for 80% [3, 10, 14, 15]. Among 295,000 maternal deaths due to preventable causes, PPH contributes to 27.1% of maternal deaths, the majority of which occur within four hours after childbirth [16–19].

Despite its proven benefit and the fact that lower-level healthcare providers can provide AMTSL, it is not regularly provided in many health facilities. According to the WHO’s recommendations, AMTSL was used as a critical intervention for PPH prevention and should be given to all women during childbirth by skilled healthcare providers [20, 21]. Annually, an estimated 1.4 million women do not get accurate practice of AMTSL during childbirth; this leads to lost opportunities for preventable PPH. Based on the definition by the International Federation of Gynecology and Obstetrics (FIGO) and the International Confederation of Midwives (ICM), the correct use of AMTSL was very low, with an average of 9% globally [22].

In many low-income countries (LMIC), women who start bleeding have limited or no access to life-saving maternity care. Uterine atony, which occurs when the uterine muscles fail to contract after birth, is the most common cause of PPH and can be prevented in most cases by using the evidence-based clinical practice of AMTSL. Despite this, AMTSL is not regularly practiced in many health facilities around the world, a significant quality issue in maternity care [23].

Globally, 295 000 maternal deaths occurred in 2017, and from those, 196,000 women died in sub-Saharan Africa (sSA). SSA and Southern Asia accounted for approximately 86% (254 000) of the estimated global maternal deaths in 2017, with sSA alone accounting for 66% with a maternal mortality ratio (MMR) of 542 per 100,000 live births [24].

In Ethiopia, nearly 1.3 million women get pregnant each year; only 26% of them are assisted by skilled birth attendants. A community-based cross-sectional study in Bale, Ethiopia revealed that only 29.2% were assisted by skilled birth attendants [25]. Other studies in Ethiopia revealed rates of AMTSL ranging from 16.7% to 32.3% [26–28]. Such low rates lead to many lost opportunities to prevent PPH [29].

AMTSL practices varied by institution type, educational level of health care providers and availability of guidelines [22, 29–32]. In addition to care-related factors, sociodemographic factors, exposure to training and work experience were significantly associated with good practice of AMTSL. To our knowledge, no studies have been conducted in Eastern Ethiopia. Moreover, this study includes different factors, which were not addressed in the previous Ethiopian studies, such as the presence of skilled birth attendants and the availability of functional fridges. Thus, this study aimed to assess the practice of AMTSL and associated factors among maternity care providers in public health facilities in Eastern Ethiopia.

## Methods

### Study setting and population

An institutional-based cross-sectional study was conducted in Harari regional state, Dire Dawa city administration and East Hararge zones of the Oromia Regional state, Eastern Ethiopia from July 15 to October 30, 2021. All MCPs who worked in maternity units in public health facilities of Harari, Oromia regional states and Dire Dawa city administration were included in the study. Senior obstetricians and gynecologists were excluded from the study because they generally are not present during the first 1–2 h of the postpartum period and they are only concerned with complicated labor.

**Fig. 1:**
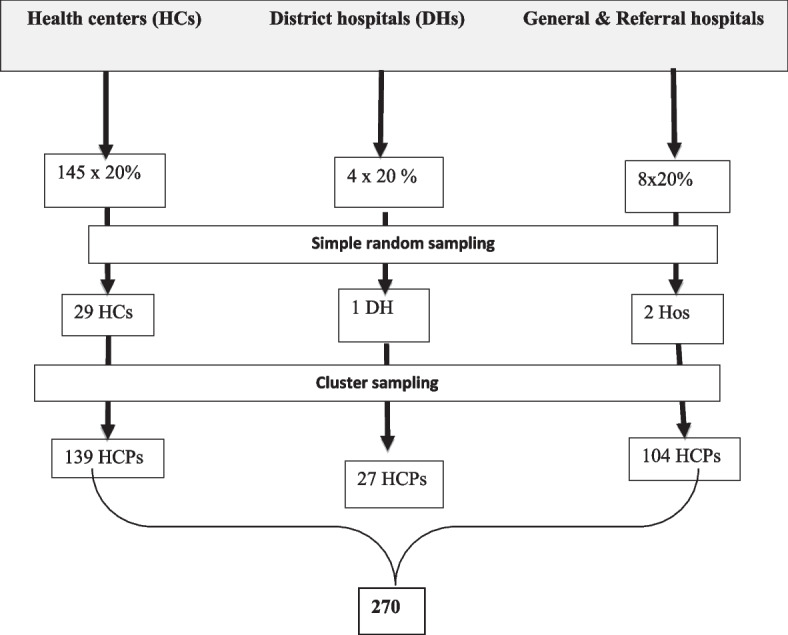
Schematic presentation of the sampling procedure

Primarily 32 health facilities (29 health centers and 3 hospitals) were selected randomly. Then MCPs were selected by using clusters in those facilities. The minimum sample size was determined by taking the proportion of good practice of AMTSL from the previous study 16.7% into account [26, 33, 34]. Since we used a cluster of MCPs in the selected health facilities, all 270 MCPs were considered as the final sample size (Fig. 1).

### Data collection methods and quality control

Data were collected through a self-administered questionnaire and using an observation checklist derived from FIGO/ICM guidelines [6, 35]. The questionnaire consisted of three sections: sociodemographic characteristics, facility-related factors, and knowledge-related characteristics. The questionnaire included 27 items for the participants to respond and the checklist included a total of 19 items filled in by the data collectors. A total of 17 midwives collected the data and nine of them were graduates with diplomas in midwifery and eight were bachelor graduates of midwifery. Six bachelor graduates of midwifery with previous experience in supervision of data collection were recruited as supervisors. These were assigned, to facilities, where they themselves did not work. The data collectors used an observational checklist to observe the practice of AMTSL. Just before the observation of birth attendants started, data collectors were familiarized with the birth attendants. The observation was non-participatory while using the checklist. A checklist was also used to assess the availability of equipment and supplies.

Training about data collection tools, ethical issues, and the quality of data was given to data collectors and supervisors. Before actual data collection, the observational checklist and questionnaires were pretested on 5% (14 MCPs) of the sample size in the Somali region (Karamara General Hospital). To minimize the Hawthorne effect during the observation of AMTSL, birth attendants were told no name will be mentioned and they were not aware that the third stage of labor was our main observation area. The filling of the questionnaire was checked by the principal investigator and supervisors daily.

### Measurements and operational definitions

#### AMTSL

Includes I). Administration of 10 IU of oxytocin (the drug of choice) via intramuscular (IM) injection within one minute after the birth of the baby. II). CCT, with gentle traction of the cord and manual support to the uterus III). Immediate uterine massage following delivery of the placenta and palpation of the uterus to assess the need for continued massage every 15 min for 1–2 h following childbirth [6].

#### CCT

The application of gentle traction of the umbilical cord, with upward, manual support of the uterus, as a delivery method for the placenta [6].

#### Practice

Is defined as an MCP's ability to carry out AMTSL.

#### Good practice

During observation, a caregiver who performed all the following: Administered the right dose of oxytocin within one minute after childbirth, deliver the placenta using CCT, immediate massage uterine fundus and continue massage every 15 min for the first 1–2 h after childbirth [27, 36–39].

### Data processing and analysis

First, data were controlled manually for completeness and consistency. Each questionnaire was assigned a unique code and entered into Epi-Data version 4.6 and analyzed by SPSS version 25. Frequencies, proportions, and summary statistics were used to describe the characteristics of study participants and findings were presented in tables and graphs. Multicollinearity was checked using standard error (0.127). The Hosmer Leme-show test (0.307) and the omnibus test (*p* = 0.000) were performed to test for model fitness. Bivariable logistic regression was carried out to study the association for each independent variable with the practice of AMTSL. Variables with *p*-values < 0.25 were taken into the multivariable logistic regression model. aOR with 95% CI was used to show the direction and strength of the association. Finally, those variables with *p*-values ≤ 0.05 were considered statistically significant.

## Results

### Sociodemographic characteristics

A total of 258/270 MCPs (95.6%) participated in the study. Mean (± SD) age was 28.49 (± 4.11) years and 198 (76.7%) were in the age group of 20–30 years. Of the total participants, 166 (64.3%) were females, 155 (60.1%) were married, 179 (69.4%) were of Oromo ethnicity, and 126 (48.8%) were Muslims. Profession-wise, 225 (87.2%) of the respondents were midwives and more than half (52.7%) were having bachelor’s degrees. Those with five years or less of work experience account for 154 (59.7%) (Table 1).

**Table 1:** Socio-demographic characteristics of maternity care providers in public health facilities in Eastern Ethiopia, 2021 (*n* = 258)

Variable	Categories	Frequency	Percent
Age	20–30 years	198	76.7
	> 31 years	60	23.3
Sex	Male	92	35.7
	Female	166	64.3
Marital status	Single	96	37.2
	Married	155	60.1
	Divorced	7	2.7
Educational level	Diploma	96	37.2
	Degree	136	52.7
	MSc and above	26	10.1
Profession	Nurse	8	3.1
	Midwife	225	87.2
	Health officer	4	1.55
	General practitioner	4	1.55
	Resident	17	6.6
Work experience	≤ 5 years	154	59.7
	6–10 years	85	32.9
	≥ 11 years	19	7.4

### Facility and maternity care providers’ related characteristics

Of all 258 MCPs participated, 138 (53.5%) were working in health centers. All health facilities had uterotonic drugs (10 IU oxytocin). Availability of standard documents in the ward was observed in 164 and only 78/164 (47.6%) MCPs with these standard documents performed the correct practice of AMTSL. A functional fridge in the right point of use (birthing room) for appropriate use of oxytocin was observed and 85/215 (39.5%) MCPs who work in a room with functional fridges practiced AMTSL correctly. Of 174 (67.7%) MCPs who had assistance from other workers during the third stage of labor, 99.4% of them administered oxytocin within one minute (Table 2). More proportion of MCPs in primary hospitals practice good AMTSL (63%) compared to the other levels of public health facilities (Table 4).

**Table 2:** Facility and maternity care providers’ characteristics in public health facilities in Eastern Ethiopia, 2021 (*n* = 258)

Variable	Frequency	Percent (%)
Availability uterotonic drugs		
Oxytocin		
Yes	258	100
No	0	0
Ergometrine		
Yes	117	45.3
No	141	54.7
Misoprostol		
Yes	187	72.5
No	71	27.5
Number of healthcare providers		
Referral Hospital	64	24.8
General Hospital	29	11.2
Primary Hospital	27	10.5
Health Center	138	53.5
Availability of functional fridge in the birthing room		
Yes	215	83.3
No	43	16.7
Time of oxytocin drug preparation/loading time		
Before the third stage of labor	165	64
During the third stage of labor	93	36
Availability of standard document		
Yes	164	63.6
No	94	36.4
Availability of birth assistant		
Yes	174	67.4
No	84	32.6
Attended training		
Yes	140	54.3
No	118	45.7

**Fig. 2:**
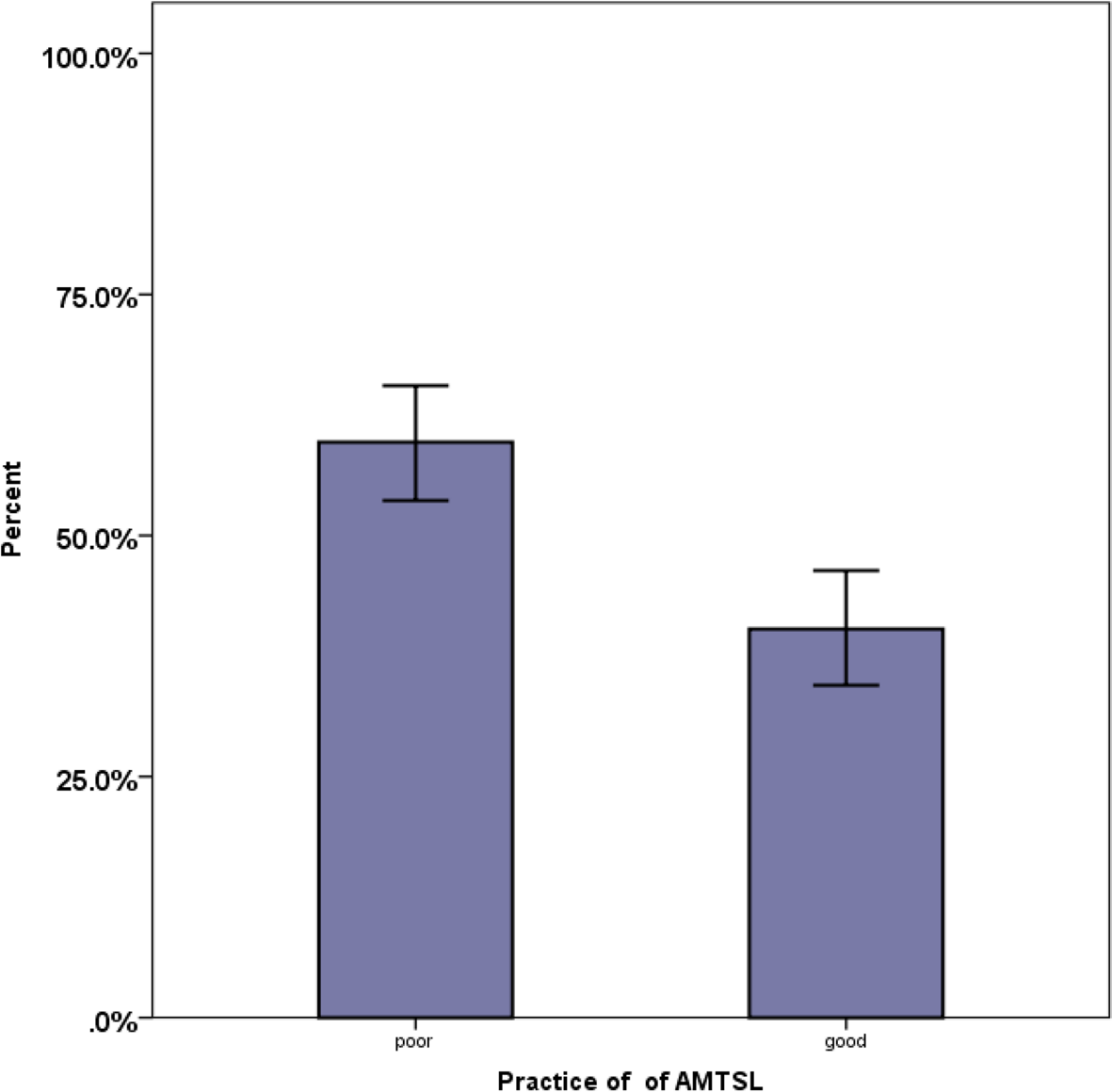
Practice of obstetric care providers on AMTSL in public health facilities in Eastern Ethiopia, 2021. The bar graph with error bars indicated that there was no overlap of the error bar lines indicating there was a statistically significant difference between the two groups at a *p*-value < 0.05

**Fig. 3:**
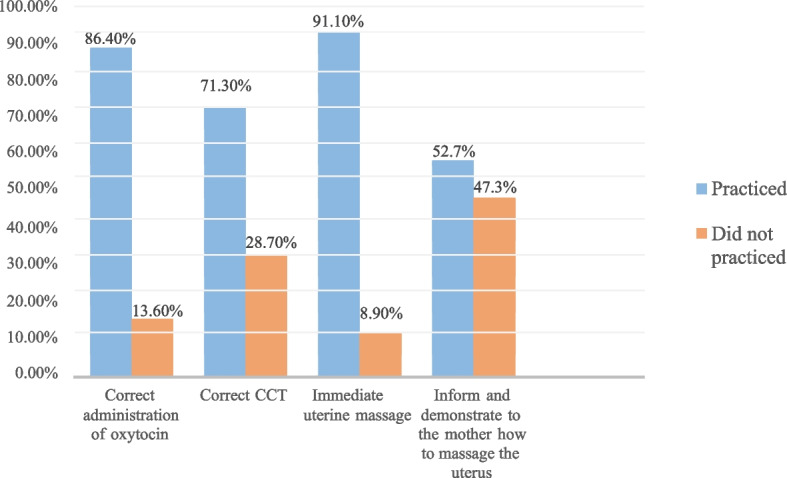
Practice of individual components of AMTSL among obstetric care providers in public health facilities in Eastern Ethiopia, 2021 (*n* = 258)

All MCPs practiced at least one of the four components of AMTSL. Only 104, 40.3% [95% CI; 34.5%-46.1%] of MCPs had good practice of AMTSL (Fig. 2). More than 90% of the MCPs practiced immediate uterine massage and 85% correct administration of Oxytocin (Fig. 3). Twenty-one (8.1%) MCPs practiced only one, 51(22.1%) at least two and 76 (29.5%) three components of AMTSL.

The majority, 204/258 (79.1%) MCPs ruled out the presence of a second baby. All the MCPs administered oxytocin 10 IU IM and 223 (86.4%) within a minute after childbirth. The majority, 154/258 (59.7%) of the MCPs did CCT within a minute. About 184/258 (71.3%) applied correct CCT. Two hundred eighteen (84.5%) MCPs assessed whether the removal of the placenta and membranes was complete. Two hundred thirty-five (91.1%) practiced uterine massage immediately after delivery of the placenta. About 52.7% of the MCPs informed and demonstrated to the mother how to massage the uterus every 15 min for the first two hours (Table 3). MCPs practiced uterine massage most frequently than other components of AMTSL as outlined by FIGO/ICM standard documents after every 15 min for 1–2 h. Inform and demonstrating to the mother how to massage the uterus was the least frequently practiced component with only 52.7% of the birth attendants and/or the mother (Fig. 3).

**Table 3:** Maternity care providers practice of active management third stage of labor in Eastern Ethiopia, 2021 (*n* = 258)

Items on checklist	Response	Frequency	Percent
Rule out the presence of a second baby	Yes	204	79.1
	No	54	29.1
Administration of oxytocin 10 IU IM	Yes	258	100
Time of oxytocin drug given within 1 min	Yes	223	86.4
	No	35	13.6
Time of cord clamped (in minutes)	< 1	154	59.7
	1–3	104	40.3
Wait for strong uterine contraction	Yes	177	68.6
	No	81	31.4
Wait for a gush of blood	Yes	101	39.1
	No	157	60.9
Correct CCT applied	Yes	184	71.3
	No	74	28.7
Placenta is supported by both hands	Yes	222	86
	No	36	14
Extract membranes gently with lateral movements	Yes	198	76.7
	No	60	23.3
Assess the completeness of the placenta and membranes	Yes	218	84.5
	No	40	15.5
Uterine massage immediately after delivery of the placenta	Yes	235	91.1
	No	23	8.9
Ensures the uterus doesn’t relax after stopping uterine massage	Yes	200	77.5
	No	58	23.5
Inform and demonstrate to the mother how to massage the uterus every 15 min for the first two hours	Yes	136	52.7
	No	122	47.3

### Bivariable and multivariable logistic regression

Variables like age, educational level, workplace, work experience, training, presence of birth assistance, availability of standard documents and knowledge about the AMTSL had *p*-values ≤ 0.25 on bivariate analysis and were included in the final model, multivariable logistic regression.

MCPs who had the highest education levels (masters and above) were 4 times more likely to perform good practice of AMTSL than those with lower-level education (diploma levels) [aOR 4.21; 95% CI 1.08, 16.40]. The odds of good practice on AMTSL were 3 times higher among those who had training on AMTSL compared to those who did not [aOR 3.02; 95% CI 1.60, 5.70]. MCPs who managed the third stage of labor with assistance were 3 times more likely to demonstrate the good practice of AMTSL than those who managed the third stage alone [aOR 2.9; 95% CI 1.42,6.04]. MCPs) having good knowledge of AMTSL were 3 times more likely to have good practice of AMTSL than their counterparts [aOR 3.00; 95% CI 1.45, 6.20] (Table 4).

**Table 4:** Bivariate and multivariable logistic regression of factors associated with the practice of AMTSL among maternity care providers in Eastern Ethiopia, 2021

Variables	Category	Practice of AMTSL		OR (95% CI)	
		Good	Poor	cOR	aOR
Age in years	20–30	70 (35.4%)	128 (64.6%)	**1**	
	> 30	34 (56.7%)	26 (43.3%)	2.4 (1.33, 4.31)	1.27 (0.53, 3.00)
Educational level	Diploma	25 (26%)	71 (74%)	**1**	
	Degree	60 (44.1%)	76 (55.9%)	2.24 (1.27, 3.96)	1.36 (0.64, 2.92)
	MSc and above	19 (73.1%)	7 (26.9%)	7.71 (2.90, 20.52**)**	4.21 (1.08,16.40) *
Workplace	Referral Hosp	31 (48.4%)	33 (51.6%)	2.22 (1.21, 4.10)	0.59 (0.21, 1.69)
	General Hosp	15 (51.7%)	14 (48.3%)	2.54 (1.12, 5.73)	1.55 (0.48, 5.00)
	Primary Hosp	17 (63%)	10 (37%)	4.02 (1.7, 9.53)	2.28 (0.72, 7.19)
	Health center	41 (29.7%)	97 (70.3%)	**1**	
Work experience	< 5 years	47 (30.5%)	107 (69.5%)	**1**	
	6–10 years	46 (54.1%)	39 (45.9%)	2.69 (1.55, 4.64)	1.58 (0.77, 3.21)
	> 11 years	11 (57.9%)	8 (42.1%)	3.13 (1.18, 8.28)	2.32 (0.61, 8.85)
Training	Yes	76 (54.3%)	64 (45.7%)	3.8 (2.23, 6.54)	3.02 (1.60, 5.70) **
	No	28 (23.7%)	90 (76.3%)	**1**	
Presence of assistance during 3^rd^ stage of labor	Yes	87 (50%)	87 (50%)	3.94 (2.14, 7.25)	2.9 (1.42, 6.04) **
	No	17 (20.2%)	67 (79.8%)	**1**	
Availability of standard document	Yes	78 (47.6%)	86 (52.4%)	2.37 (1,37, 4.10)	1.28 (0.51, 3.23)
	No	26 (27.7%)	68 (72.3%)	**1**	
Knowledge	Good	83 (53.9%)	71 (46.1%)	4.62 (2.6, 8.2)	3.00 (1.45, 6.20) **
	Poor	21 (20.2%)	83 (79.8%)	**1**	

Significant at p* < 0.05, p** < 0.01, 1 = Reference

## Discussion

Overall good practice of AMTSL was observed in 40.3% [95% CI: 34.5%-46.1%] of MCPs. This implies that 60% of laboring mothers might face life-threatening complications like severe hemorrhage and its related complications. This finding is consistent with previous studies in Ethiopia (Tigray 43.3%) and Nigeria (41.7%), but higher than studies in Sudan (26.7%), and Ethiopia such as in Kembata Tembaro zone (29.8%), Amhara region (32.3%), Sidama zone (32.8%) and Hawassa city (15.7%) [26–28, 38, 40–42]. The disparity could be attributed to the study period, educational level, and training. Only 20% of participants in Sudan were trained and all had a diploma and less than three years of experience; in Sidama, only 29% of participants were trained, 74% had diplomas and 91% worked in health centers; and in Hawassa, only 33.3% were trained, 75% had diplomas and 33% had good knowledge of AMTSL. In our study, however, more than half of the participants were trained, had good knowledge of AMTSL, had master's degrees or higher and 40% had more than six years of experience.

On the other hand, good practice of AMTSL was lower than in other studies in Ethiopia such as Amhara (61.2%), Gamo Gofa (48.1%) and Addis Ababa (47%) [37, 39, 43]. In the Gamo-Gofa study, the same provider was three times observed to assess AMTSL practice, while in our study observations were only once, levelling out the possibility of learning during the first and second observations. In Amhara, different methodological issues (tools and parameters used) as means were used as a cut-off point to indicate good AMTSL practice. In some studies, only referral hospitals were included instead of all levels of public health facilities, In Addis Ababa only convenience sampling was used.

In our study, having the highest educational level, being trained, the presence of a birth assistant, and having good AMTSL knowledge were statistically associated with AMTSL practice.

Training was statistically significantly linked to good practice of AMTSL, supported by studies in Kenya and Ethiopia [31, 36, 39, 44, 45]. AMTSL training may allow them to demonstrate actual practice and recall this when performing AMTSL in a real-world setting. Furthermore, training updates knowledge about AMTSL components, which influences actual practice toward AMTSL.

Good knowledge was more likely to result in good AMTSL practices in line with studies in Kenya, Nigeria, and Ethiopia [27, 36, 38, 39, 43]. Knowing the definition of AMTSL and its components, as recommended by ICM/FIGO, increases the likelihood of AMTSL implementation with more motivation to put knowledge into practice leading to improved performance.

Presence of birth assistants in addition to MCPs increased the likelihood of good practice of AMTSL. This is in harmony with studies in Ethiopia and Nigeria [28, 38]. The presence of assistants is one of the critical factors in performing good AMTSL and this is true in all maternity procedures where only team efforts will provide adequate care.

Furthermore, in the current study, MCPs with the highest educational level were more likely to practice good AMTSL, in harmony with other studies in Ethiopia, specifically in Addis Ababa and Tigray [39, 40].

## Strengths and limitations

### Strength

- The tool used to collect data was adapted from validated sources and pretested
- Direct observation of’care providers’ practice (observational checklist)
- Included all public health facilities in three different administrative areas

### Limitations

- Hawthorne effect (the practice of MCPs was investigated by direct observation, which might change the observed behaviors, or knowing their performance is evaluated, could affect the results)
- The cross-sectional nature of the study design is not allowing a cause-and-effect relationship.

## Conclusions and recommendations

Overall good practice of AMTSL was relatively low. Two in every five MCPs correctly performed AMTSL. Immediate uterine massage and administration of oxytocin injections were the most practiced components of AMTSL, much more frequently than subsequent uterine massage.

Being trained, higher educational level, having birth assistance and having good knowledge of AMTSL were statistically significantly associated with good practice of AMTSL.

It is important to organize seminars and workshops on AMTSL to update knowledge through consistent and sustainable pre-service and in-service training that directly will improve practices of good AMTSL.

## Data Availability

The data sets used for this study are available from the corresponding author (DT) at reasonable request.

## Abbreviations

AMTSL: Active Management of the Third Stage of Labo
aOR: Adjusted Odds Ratio
CCT: Controlled Cord Traction
CI: Confidence Interval
cOR: Crude Odds Ratio
FIGO: Federation International of Gynecology and Obstetrics
ICM: International Confederation of Midwives
MCPs: Maternity Care Providers
PPH: Postpartum Hemorrhage
